# State-of-the-Art Review: Managing Vulvovaginal Candidiasis

**DOI:** 10.1093/cid/ciaf673

**Published:** 2026-03-17

**Authors:** Riina Rautemaa-Richardson, Jack D Sobel, Neil Stone, Francesco De Seta, Antonio Cassone, Pedro Vieira-Baptista, Manola Comar, Adilia Warris, Elena Roselletti

**Affiliations:** Mycology Reference Centre Manchester and Department of Infectious Diseases, ECMM Centre of Excellence, Manchester Academic Health Science Centre, Wythenshawe Hospital, Manchester University NHS Foundation Trust, Manchester, United Kingdom; Division of Evolution, Infection and Genomics, Faculty of Biology, Medicine and Health, University of Manchester, Manchester, United Kingdom; Department of Internal Medicine, Wayne State University School of Medicine, Detroit, Michigan, USA; Hospital for Tropical Diseases/University College London Hospitals Infection Division, London, United Kingdom; Centre for Clinical Microbiology, Division of Infection and Immunity, University College London, London, United Kingdom; Department of Obstetrics and Gynaecology, IRCCS San Raffaele Scientific Institute, Milan, Italy; Polo d'Innovazione della Genomica, Genetica e Biologia, Siena, Italy; Faculty of Medicine, Department of Gynecology-Obstetrics and Pediatrics, Universidade do Porto, Porto, Portugal; Department of Human Structure and Repair, Faculty of Medicine and Health Sciences, Ghent University, Ghent, Belgium; Department of Gynecology and Obstetrics, Hospital Lusíadas Porto, Porto, Portugal; Advanced Translational Microbiology, Institute for Maternal and Child Health-IRCCS Burlo Garofolo, Trieste, Italy; Department of Medicine, Surgery and Health Sciences, University of Trieste, Trieste, Italy; Department of Biosciences, Faculty of Health and Life Sciences, Medical Research Council Centre for Medical Mycology at the University of Exeter, Exeter, United Kingdom; Department of Biosciences, Faculty of Health and Life Sciences, Medical Research Council Centre for Medical Mycology at the University of Exeter, Exeter, United Kingdom

**Keywords:** vulvovaginal candidiasis, recurrences, *Candida*, vaginal microbiome, antifungal and emerging therapies

## Abstract

Vulvovaginal candidiasis is one of the most prevalent infections in women worldwide. Together with its recurrent form, it affects millions of women annually, causing significant symptoms and severely impacting quality of life. This review examines the pathophysiology, risk factors, microbiome interactions, clinical manifestations, and challenges in diagnosing and managing vulvovaginal candidiasis, with emphasis on recurrent vulvovaginal candidiasis. While *Candida albicans* is the primary cause, non-*albicans* species are increasingly common. Multiple factors contribute to both forms, including hormonal changes, diabetes, antibiotic use, immune dysfunction, and genetics. The vaginal microbiome plays a key role in maintaining homeostasis and preventing *Candida* overgrowth. Symptoms such as itching, discharge, and soreness overlap with other conditions, complicating the diagnosis. Standard treatment involves topical or systemic antifungals, but recurrence and resistance are frequent. Emerging strategies include novel antifungals, immunomodulators, and vaccines. Future approaches should focus on modulating host and environmental factors to prevent recurrence, reduce resistance, and improve outcomes.

## Teaser

Vulvovaginal candidiasis (VVC), especially in its recurrent form, significantly impacts women's health. This review explores its pathophysiology, risk factors, microbiome interactions, diagnosis, management, and treatment, emphasizing emerging strategies such as new drugs, zinc supplementation, and immunotherapy beyond conventional antifungal therapies.

**Figure:**
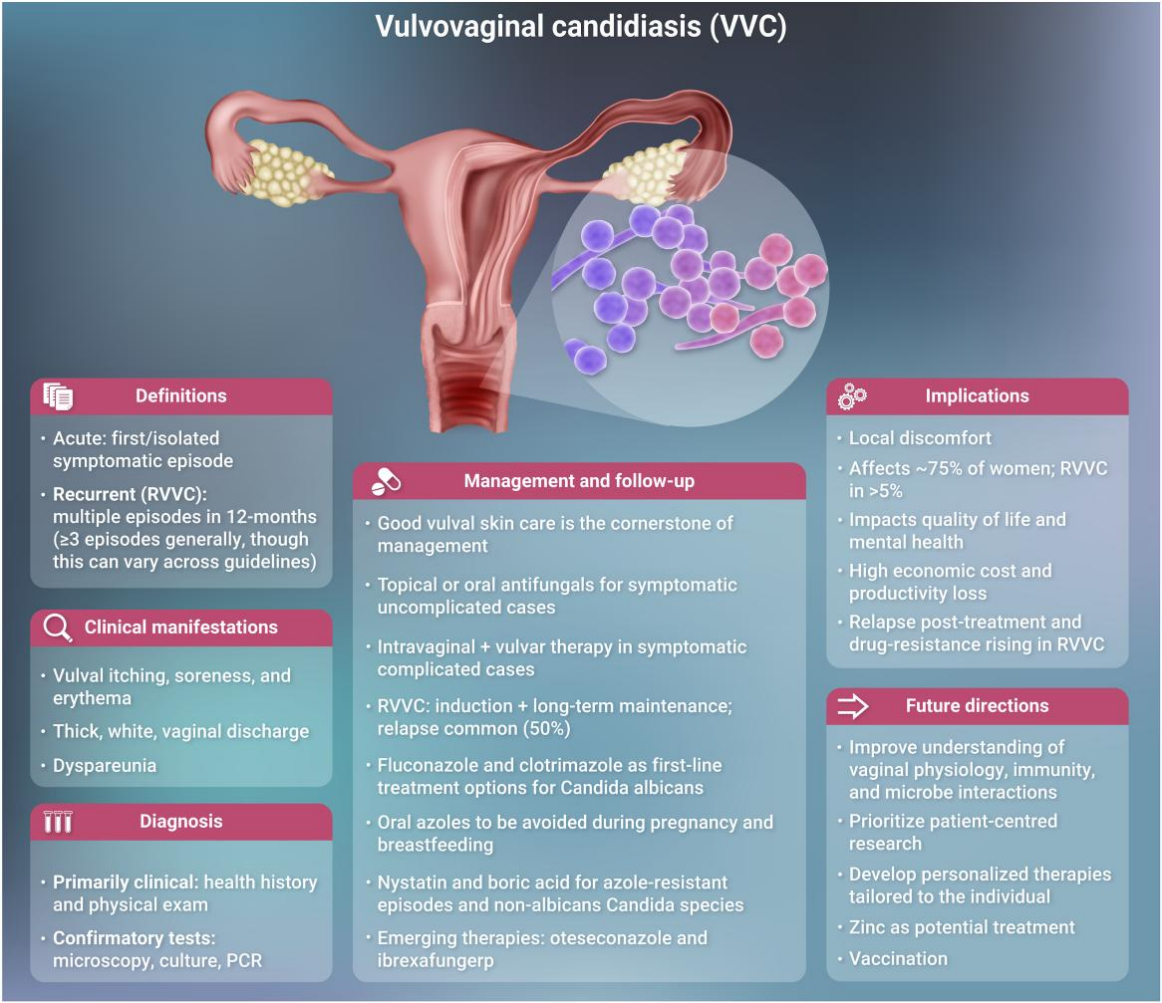


Acute vulvovaginal candidiasis (VVC) is among the most prevalent infections in women worldwide, especially in those of reproductive age. Acute VVC and its recurrent form (RVVC) affects an estimated 138 million women every year [1]. It not only causes significant clinical symptoms and disease but also severely impacts women's quality of life (eg, sexual, mental health) [2]. Understanding the complexity and burden of these infections is essential for improving diagnostic, therapeutic, and preventive strategies. VVC is a common fungal infection of the female genital tract, primarily caused by *Candida* species [2]. In this review, the species *Candida albicans* is referred to explicitly. For other pathogenic species, we use the historical term non*-albicans Candida* (NAC) for simplicity; however, the currently accepted genus and species names are provided for all other pathogenic species causing VVC, in accordance with recent taxonomic revisions. Approximately 75% of women experience at least 1 acute episode of VVC in their lifetime [2], with 5%–10% developing RVVC, defined as 3 or more [3] symptomatic episodes of VVC within a 12-month period. Although most cases occur in reproductive-aged women, VVC can also affect adolescents and postmenopausal women [4, 5].

The condition is characterized by vulval symptoms such as pruritus, erythema, fissures, and dysuria as well as vaginal discharge and dyspareunia, which can impair daily activities and quality of life [1, 2]. Beyond the physical complaints, VVC and RVVC are associated with psychosocial distress, including increased anxiety, depression, and social isolation [2]. The economic impact is substantial, with annual treatment costs exceeding $368 million in the United States and global productivity losses estimated at approximately $14 billion [2, 6].

Although *C. albicans* remains the predominant pathogen responsible for the majority of VVC cases, infections caused by NAC species, such as *Candida glabrata* (*Nakaseomyces glabratus*)*, Candida krusei* (*Pichia kudriavzevii*)*, Candida parapsilosis*, and *Candida dubliniensis,* are increasingly reported, particularly in developing countries where their prevalence ranges from 21% to 72% [7]. These infections are more common among patients with a history of multiple courses of fluconazole treatment and in patients with diabetes [8–10]. Although NAC species are often less responsive to first-line antifungal therapies, *C. albicans* remains susceptible to first-line antifungals [9]. NAC prevalence is particularly high in settings with limited access to healthcare and diagnostics, poorly controlled sales of pharmaceuticals, and lack of antimicrobial stewardship activities [1].

There are 2 major gaps that need to be addressed to reduce the global health and economic burden of RVVC. The first is the global disparity in access to healthcare and diagnostics leading to emergence of antifungal resistance hot spots with limited management options [11]. The second is a lack of patient education and overmedicalizing vulval complaints [12]. Furthermore, social and cultural barriers may also contribute. Shame, stigma, and fear of judgment, well documented in other areas of sexual and reproductive health, may similarly discourage some women from seeking timely care for vulvovaginal symptoms [13, 14]. This is particularly the case if they believe that RVVC is a sexually transmitted infection, and education plays a key role in clarifying this misunderstanding. Educational efforts should also focus on maintaining vulval and vaginal health, including daily local skin care to help prevent and manage infection, while avoiding unnecessary, heavily advertised and broadly available feminine hygiene products. Women should have easy access to decision tools for self-diagnosis, guidance on balanced and evidence-based self-care, and faster access to clinical and laboratory diagnostic tests. Antifungal treatment should only be used in patients with symptoms in keeping with those of VVC, and patients should be always encouraged to consult an appropriate specialist to seek medical advice, especially when first-line management attempts fail.

## PATHOPHYSIOLOGY

The human vagina is a unique environment due to its distinct microbiome composition in which the microflora is dominated by lactobacilli. This maintains high lactic acid concentrations (55–111 mM), resulting in an acidic pH (3.5–4.7) [15, 16]. Together, this specific microbiome composition and lactic acid concentrations are unlike those found in any other human body site or in the vagina of other mammals and nonhuman primates [16]. This acidic environment is protective against many potential pathogens, whereas *Candida* spp. are acid-tolerant and can persist under these conditions [17].

Asymptomatic vaginal colonization by *Candida* is common, occurring in up to 20%–30% of healthy women, suggesting that these fungi may represent part of the normal vaginal microbiome [18, 19]. Consequently, microbiome disruptions, caused by factors like antibiotic use, hormonal fluctuations, or sexual activity, can lead to fungal overgrowth [20], and increase the risk of developing symptomatic VVC infections [21–23]. The interplay between fungal and bacterial pathogens in vaginal mixed and co-infections can, in some cases, exacerbate symptom severity and complicate treatment [23].

**Figure 1.**
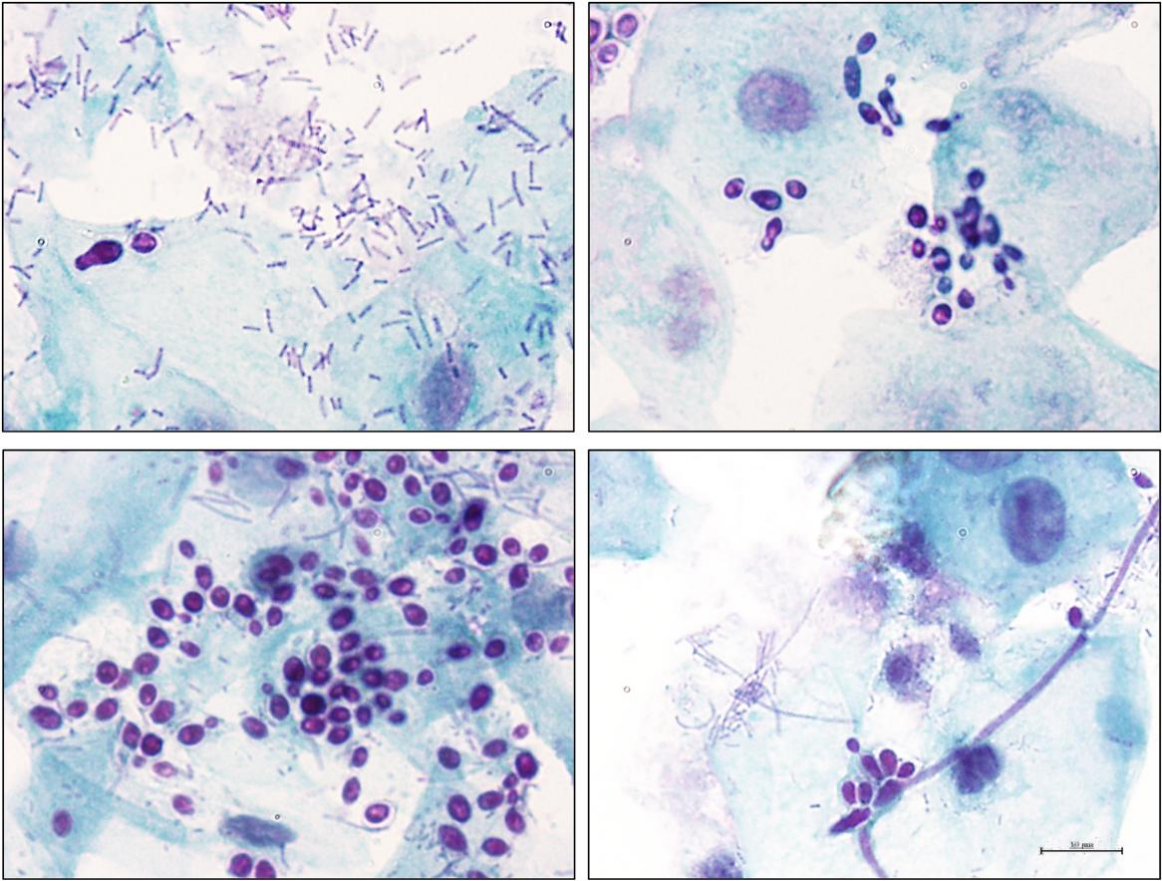
Micrographs of vaginal smears from women with or without VVC symptoms and positive for *Candida albicans* by culture highlighting the challenges of laboratory diagnosis of VVC. Vaginal swabs from 2 asymptomatic (top row) and 2 symptomatic (bottom row) women, were collected at the University Hospital Santa Maria della Misericordia, Perugia, Italy (VAG1 n. 2652/15). Samples were stained using the Papanicolaou technique and visualized at 1000× magnification (scale bar = 10 μm). Abbreviation: VVC, vulvovaginal candidiasis.

The pathophysiology of VVC is a complex interplay between fungal virulence factors and the host immune response [24]. *C. albicans* is a polymorphic fungus capable of transitioning between yeast, pseudo-hyphal, and hyphal forms, a process known as filamentation [25]. This morphological plasticity is tightly linked to its pathogenicity, as filamentation promotes tissue invasion and the expression of key virulence factors responsible for establishment of infections [25, 26] (Figure 1). Several well-characterized virulence factors, including the pH-regulated antigen 1 protein (Pra1), candidalysin, and secreted aspartyl proteinases (SAPs), may contribute to the ability of *C. albicans* to persist in the vaginal environment [27]. During infection, *C. albicans* releases Pra1, which facilitates fungal survival by scavenging zinc, an essential micronutrient within the vaginal environment. At the same time, it promotes the recruitment of polymorphonuclear neutrophils (PMNs) and plays a pivotal role by triggering a robust host inflammatory response [28]. However, despite the accumulation of PMNs in the lumen of the vaginal mucosa, fungal clearance is impaired [29]. One hypothesis is that the vaginal environment in VVC directly induces PMN dysfunction [29]. Heparan sulfate, a proteoglycan found on mammalian cell surfaces [30], has been identified as a potential contributor to PMN dysfunction in in vitro and experimental models, and its binding to CD11b on PMNs results in impaired reactive oxygen species production and NETosis (the release of neutrophil extracellular traps, networks of DNA and antimicrobial proteins that trap and kill pathogens), thereby reducing *C. albicans* clearance [31, 32]. Estrogen may exacerbate this process by increasing heparan sulfate expression, and it has been shown in the same experimental models that heparinase III can restore PMN activity in VVC-susceptible mice [31]. Additionally, PMN hyperactivation, triggered by fungal virulence factors such as Pra1, candidalysin, and SAPs, can cause tissue damage and vulvovaginal inflammation through excessive NET formation, protease release, and reactive oxygen species production [27–29]. Although these host responses are meant to neutralize *C. albicans*, their release may result in tissue injury rather than effective pathogen clearance. This hyperactivation, compounded by the presence of markers like perinuclear antineutrophil cytoplasmic antibodies, contributes to the inflammatory damage observed in VVC, suggesting a misalignment in the timing and location of PMN responses, worsening the infection [29, 33]. In contrast to *C. albicans* pathology, NAC species, particularly *C. glabrata*, the second most common species causing VVC [9], elicit a weaker inflammatory response and reduced PMN recruitment, which partially explains the less symptomatic presentation in these infections [28]. Much of the current knowledge on VVC pathology is based on studies using cell lines and mouse models, which, although useful, do not accurately mimic the acidic vaginal environment seen in human VVC [27, 28]. Further research is needed to better understand the pathophysiology of vulvovaginal infections, with the goal of improving current therapies and developing new targeted approaches to restore microbial balance and prevent pathogens overgrowth and recurrence.

## PREDISPOSING CONDITIONS AND RISK FACTORS

Although the reasons some women are particularly susceptible to RVVC are not fully understood, several risk factors have been identified.

### Skin Health

One of the main modifiable risk factors for VVC is dry and damaged vulval skin caused by overwashing and unnecessary use of feminine products that disrupt the skin barrier function and its microbiome. Washing (even with water) dries the skin and dry skin is more likely to be itchy. It is also more vulnerable and prone to infection. Guidelines recommend daily liberal use of an emollient cream as a soap substitute, moisturizer, and a barrier cream, and stopping the use of any other products in the area [34]. See Key talking points with patients (Table 1).

**Table 1.** **Key Talking Points With Patients From**  https://mrcm.org.uk/wp-content/uploads/2024/11/General-Care-of-the-Vulval-Skin-Information-Leaflet.pdf

Healthy skin is essential for preventing and fighting infections. The following general advice on vulval skin care may help keep the vulva healthy. vulval skin is thin and fragile and should be cleansed with an emollient cream (eg, hydromol ointment, diprobase, doublebase, cetraben) instead of soap.		
	Do	Don’t
Washing	Use emollient creams as soap substitutes. Use your hands to clean your vulva. Wash only once a day. Overcleaning can make symptoms worse. Dab dry with a soft towel.	Don’t use soaps, bubble baths, or shower gels. Avoid “feminine” washes and sprays. Avoid washing the vulva with sponges, or cloths. Do not wash inside the vagina (douching). Avoid washing your hair while sitting in the bath.
Clothes and laundry	Wear cotton underwear. Wash underwear with nonbiological products.	Avoid wearing synthetic fabrics. Avoid wearing tight clothing. Avoid wearing underwear when sleeping. Avoid fabric conditioners and biological washing powders.
Toileting, periods and sex	Use tampons or use Moon Cups during your periods instead of pads (if possible). Wipe from front to back on the toilet. Use water-based lubricants for sex.	Don’t use sanitary pads or panty liners daily. Avoid shaving or waxing your pubic hair, trimming is better.
General: Avoid scratching. This damages the skin and makes it sore. Antihistamines (anti-allergy) tablets over the counter can be taken for severe itching		

### Hormonal Influence

Elevated estrogen levels, whether endogenous during pregnancy or luteal phase of the cycle, or exogenous from combined oral contraceptives or menopause hormone therapy, enhance *Candida* spp. colonization by increasing glycogen production in the vaginal epithelium and upregulating epithelial receptors that facilitate fungal adhesion and growth [35]. Beyond glycogen metabolism, estrogen promotes filamentation and upregulates mannose-binding adhesins on *C. albicans*, contributing to persistent colonization [35]. Hormonal fluctuations, particularly during the luteal phase, or in the form of combined contraceptive pill are also associated with RVVC episodes [5]. However, progestin-only contraceptive pill or hormonal (levonorgestrel-releasing) intrauterine contraceptive devices are rarely associated with increased risk, although the intrauterine contraceptive device itself can contribute to recurrent VVC through a “foreign body” effect by facilitating fungal colonization on the device [36, 37].

### Immunosuppression and Diabetes

Conditions like untreated human immunodeficiency virus (HIV) and AIDS increase VVC risk due to T-cell dysfunction. Women with HIV show higher symptom prevalence, especially when CD4 counts are low [38, 39]. Effective antiretroviral therapy can markedly mitigate this risk [40]. In addition to causing immune dysfunction, uncontrolled diabetes elevates vaginal glucose levels promoting yeast overgrowth and proliferation [41]. At the same time, SLGT2 inhibitors used in the management of diabetes increase the risk for RVVC as they block the reabsorption of glucose in the kidneys, allowing excess sugar to be excreted in urine [42]. Various immunosuppressive therapies, including corticosteroids, chemotherapy, calcineurin and tumor necrosis factor-α inhibitors, antimetabolites, and monoclonal antibodies, compromise immune surveillance, impair mucosal integrity, and often also elevate glucose levels. Together, these effects create a favorable environment for *Candida* spp. proliferation, increasing the risk of VVC [35, 43–45].

### Antibiotics

Antibiotic use is a major contributor to VVC by disrupting the protective vaginal microbiota, especially *Lactobacillus* species. Bacterial vaginosis, an imbalance of the vaginal microbiota, characterized by a marked reduction in lactobacilli and dominance by anaerobes, may predispose women to VVC, either independently or following antibacterial treatment [23, 35].

### Environmental Factors and Diet

Tight synthetic clothing may trap heat and moisture, encouraging yeast growth. One study observed VVC symptom improvement with fibroin underwear compared to cotton [46]. Dietary factors, particularly high sugar intake, has been linked to VVC development, but mainly in patients with diabetes. Though evidence in those without diabetes is limited, some reports suggest dietary changes (eg, ketogenic diets) may reduce frequency of RVVC [47, 48]. Vitamin D appears to contribute to mucosal health and supplementation is recommended where exposure to sunlight is limited [49, 50].

### Sexual Activity

Although not a direct cause, sexual activity may expose the deeper vulvovaginal tissues to colonizing *Candida* spp. and increase VVC susceptibility [51]. VVC is not classified or considered a sexually transmitted infection, transmission between partners is rare, and partner treatment is not recommended [35, 52].

### Genetic Factors

RVVC is associated with an excessive, dysregulated immune response to low levels of colonizing *Candida* spp. Polymorphisms in genes encoding Toll-Like Receptors or Mannose-Binding Lectin impair innate antifungal responses and have been associated with reducing mucosal clearance of *Candida* [53–55]. Genetic predisposition may also play a role. Whole-exome sequencing of 160 women has linked SIGLEC15 polymorphisms to RVVC susceptibility [56, 57]. Some women with RVVC exhibit localized immune hypersensitivity, which can be associated with an exaggerated Th2-type immune response, rather than protective Th1 immunity, leading to inflammation without effective pathogen elimination [58, 59].

### Vaginal Microbiome

The human vaginal microbiome is still being unraveled, and even defining what constitutes “normal” in this field remains controversial [60]. Normality cannot be based solely on absence of symptoms. Also, the vaginal microbiome goes through significant changes with age as a response to hormonal changes. Traditionally, lactobacilli dominance (especially with species like *Lactobacillus crispatus, Lactobacillus gasseri,* or *Lactobacillus jensenii*) and low α diversity (a microbiome dominated by only a few species rather than being highly diverse) are considered optimal for women in child-bearing years [61]. This is in sharp contrast with other mammals, where lactobacilli dominance does not occur and microbial diversity is the norm [16, 62].

The variations in the incidence of VVC and colonization across a woman's lifespan are closely tied to estrogen levels and glycogen availability. These, in turn, influence the abundance of lactobacilli (higher during pregnancy and lower during childhood, breastfeeding, and menopause), and vaginal pH, which is inversely correlated with the number of lactobacilli. Clinically, women with RVVC often report symptom flares during the luteal phase and relief during menstruation. Although lactobacilli are generally viewed as protectors of the genital tract, their role in VVC is complex. Although lactic acid production and acidification inhibit many pathogens, *Candida* spp. tolerate low pH [17]. In fact, *Candida* spp. can metabolize lactate to fuel hyphal growth and tissue invasion [63, 64]. Conversely, *L. crispatus* may inhibit *C. albicans* adhesion to epithelial cells [65], and some *Lactobacillus* strains have shown the ability in vitro to suppress fungal growth, limit virulence factor expression, and block the yeast-to-hyphae transition [66, 67]. In animal models, lactobacilli also appear to protect against VVC development. However, epidemiological studies in humans often show an association between VVC (and RVVC) and lactobacilli dominance [68–70]. Some authors report that, despite preserved lactobacilli numbers in women with RVVC, there is increased diversity (eg, *Lactobacillus iners* dominance) and decreased presence of *L. crispatus* and *L. gasseri* [71–73]. The role of *L. iners*, especially in recurrent VVC, may involve its part in L-glutamate production [73]. These findings suggest that lactobacilli's protective effect against candidiasis may depend on specific species, or even strains, rather than the genus. This might explain why antibiotic use can predispose to VVC, potentially by disrupting *L. crispatus* dominance and favoring *L. iners* [74]. The fragile balance of protective lactobacilli may also help to explain why probiotics have largely yielded inconsistent or negligible results in preventing or treating VVC, as introducing additional strains may not reliably restore the optimal microbiome composition. Despite these uncertainties, it can be speculated that the role of lactobacilli as a protective factor for candidiasis is based on a very delicate equilibrium and is probably less critical than for other conditions, such as bacterial vaginosis or trichomoniasis. Probiotics are often marketed as a prophylactic or adjuvant measure against VVC. However, unsurprisingly, the evidence derived from the well-designed studies and with lower risk of bias do not support their use [75].

### Clinical Manifestations and Diagnosis

The clinical manifestations of VVC include vulval itching, burning, soreness, erythema, fissures, vaginal discharge, and dyspareunia. However, these are not specific to VVC and overlap with those of, for example, vulval eczema and other dermatoses (eg, lichen sclerosus), low-grade herpes simplex virus reactivation and vulvodynia. At the same time, a significant proportion of women are colonised with *Candida* spp., with counts fluctuating from day to day. This together with dual/concomitant pathologies being common provide significant diagnostic challenges. The diagnosis is primarily clinical, based on a detailed health history and physical examination, followed by confirmatory tests, including microscopy, culture, or polymerase chain reaction [34]. The more sensitive the laboratory diagnostic method (eg, polymerase chain reaction), the more likely a positive result reflects colonization rather than active VVC, and the symptoms might be caused by a different cause. Pre- and posttreatment fungal cultures can be helpful in ruling out alternative and additional diagnoses. If microbiologically effective treatment, defined as a negative culture from a specimen collected within 2–3 days after completing treatment, does not fully resolve the symptoms, an alternative or additional cause should be considered. To avoid unnecessary treatment of colonization, microbiological tests including posttreatment tests, should not be offered to asymptomatic women. Asymptomatic vaginal colonization does not require treatment, even if high number of *Candida* spp. cells are present.

The need for microbiological confirmatory tests can be questioned in cases where antifungal therapy is initiated without waiting for the result and the result makes no difference for patient care. Some guidelines therefore recommend treatment without confirmatory tests in uncomplicated cases and/or single episodes [34]. However, confirmatory tests are extremely helpful and essential in cases with poor or partial response and in RVVC [34, 52, 67].

### Management and Follow-up

The cornerstone of the management of any vulval complaint is good skin care (see Key talking points with patients, Table 1). This starts with daily liberal use of an emollient cream as a soap substitute, moisturizer, and a barrier cream, and stopping use of any other products in the area.

Clinical practices and patient histories suggest that the use of water-based lubricants during intercourse might be considered for women whose RVVC flares are triggered by intercourse. The therapeutic options include systemic and topical azoles, topical polyenes, and boric acid [34, 52, 67]. Gentian violet is still mentioned in some treatment guidelines, but its use has largely been abandoned because of poor tolerability, safety concerns, and the availability of safer and more effective alternatives [76–78].

In uncomplicated VVC, defined as mild to moderate infections that occur infrequently and respond to standard antifungal therapy, the main options with similar efficacy are oral fluconazole as a single 150-mg dose or various azole containing vaginal suppositories and creams, with treatment durations ranging from 1 to 7 days. These topical treatments are considered safe without any significant systemic absorption, whereas systemic antifungals are contraindicated in pregnancy. All established vaginal and topical antifungals are well tolerated. In cases of extensive vulval erythema, edema, or excoriation, oral treatment is often preferred because topical antifungals may be poorly tolerated. However, topical treatment provides very high local concentrations of drug to the site of infection and may provide a faster response. Combining topical intravaginal treatment with oral treatment and/or an additional antifungal cream for the vulva can also be helpful in severe cases [79]. Brief courses of mild topical steroid creams can sometimes be considered for quick symptom relief. The treatment options for NAC and azole-resistant infections include nystatin and boric acid suppositories for 1–2 weeks duration, and at least 21 days for resistant infections on specialist consultation. Both of these topical agents are well tolerated, but boric acid is contraindicated in pregnancy because of a lack of safety data (Table 2). Due to variations in doses available commercially, most scientific and safety data supports the use of 600 mg of boric acid daily per vagina in a gelatine capsule.

**Table 2.** Summarized First-line Treatment Recommendations for Vulvovaginal Candidiasis (VVC) and Recurrent VVC [3–6]

	Treatment	Comments
Acute VVC	Fluconazole 150 mg p.o. (single dose)	Not during pregnancy
	Clotrimazole 500 mg p.v. (single dose)	First line during pregnancy.
	Nystatin 100 000 IU p.v. for 14 nights	For non-*albicans Candida* spp. and azole resistance
	Ibrexafungerp 300 mg p.o. (2 doses for 1 day)	Not during pregnancy
	Boric acid 600 mg p.v. for 14–21 days	For non-*albicans Candida* spp. and azole resistance. Not during pregnancy. Significant variation of availability, strengths and products
	Oteseconazole p.o. 600 mg first dose, followed by 450 mg the following day	Not during pregnancy and contraindicated in females of reproductive potential
Recurrent VVC	Fluconazole 150 mg p.o. every 72 hours for 3 doses followed by 150 mg weekly for 6 months	Not during pregnancy
	Nystatin 100 000 IU p.v. daily for 14 nights/month for 6 months	For non-*albicans Candida* spp. and azole resistance
	Clotrimazole 500 mg p.v. for 10–14 days, followed by 500 mg weekly	First line during pregnancy
	Boric acid following induction dose, 600 mg p.v. initially daily, then 2–3 times per week for 6 months	For non-*albicans Candida* spp. and azole resistance. Not during pregnancy. Significant variation of availability, strengths and products
	Oteseconazole p.o. following induction dose, weekly 150 mg for 11 weeks	Not during pregnancy and contraindicated in females of reproductive potential

Abbreviations: p.o., orally; p.v., per vagina.

RVVC is a challenging condition and difficult to manage [5, 52, 80]. Good daily skin care and management of all modifiable risk factors are extremely important for success. The standard antifungal treatment involves induction therapy (eg, three 150-mg tablets of fluconazole 72 hours apart) followed by maintenance/suppression therapy (eg, once weekly 150 mg fluconazole) for 6 months (Table 2). It is important to manage patient's expectations when starting them on this pathway and to clarify that the purpose of the 6-month suppression is only to give them time without symptoms and quality of life without fear of recurrence, and to allow the microbiome and immune responses to settle. Given that no treatment will fully eradicate *Candida* from their body, symptoms recur in 50% of patients who stop suppressive treatment [81]. In these cases, 1 approach is to provide patients an induction treatment once more followed by suppression. Depending on the symptom-free interval, the time between treatment doses can be extended to (eg, every 2 weeks instead of weekly) or timed, taking into account when during the menstrual cycle the flares do present. This is to provide them with quality of life with minimum amount of treatment. The success of the suppression is measured by lack of symptoms during treatment rather than the interval to flare after stopping. An illustrative case description how to manage RVVC is presented in Table 3.

**Table 3.** An Illustrative Case of RVVC Caused by *C. glabrata* is Described, Followed by Approach and Treatment

Case description	A 36-year-old woman presents with RVVC. She reports experiencing VVC episodes for as long as she can remember, occurring approximately monthly. Over the years, she has consulted her family physician and received multiple treatments, including clotrimazole and fluconazole pessaries. These provided temporary relief initially but are no longer effective. A recent vaginal swab revealed heavy growth of *Candida glabrata* (*Nakaseomyces glabrata*), with a minimum inhibitory concentration (MIC) to fluconazole of 8 μg/mL.
Approach	The initial step is to identify and address any reversible causes. A comprehensive assessment of risk factors should be conducted. Pregnancy should be excluded, given that pregnancy elevates VVC risk and restricts antifungal treatment options due to potential contraindications [35]. A detailed history of antibacterial use should be obtained, with efforts to minimize unnecessary antibiotic courses, as they disrupt vaginal microbiota and promote *Candida* spp. overgrowth [82]. Medication history should explore immunosuppressant use, particularly corticosteroids, which impair antifungal immunity [43]. Lifestyle and environmental factors should also be reviewed: vaginal douching should be discouraged, although it is not an official risk factor for VVC, and clothing choices (eg, tight, nonbreathable fabrics) that may trap moisture, and heat should be discussed and modified if needed [46 ]. Referral to dermatology or gynaecology is recommended to evaluate alternative diagnoses, such as vulval dermatitis, lichen sclerosus, or lichen simplex chronicus, which may mimic VVC symptoms. The isolation of *C. glabrata* from a swab may reflect colonization rather than true infection, as *C. glabrata* rarely causes vulval disease [35].
Treatments	Antifungal treatment will likely be required, but fluconazole is expected to be ineffective against *C. glabrata* because of its reduced susceptibility [67]. Treatment options, informed by the Infectious Diseases Society of America (IDSA) candidiasis guidelines [67] and the British Association for Sexual Health and HIV (BASHH) Candida guidelines [34], are summarized in Table 2. The flow diagram (Figure 2) captures the clinical decision-making process. As a last resort, oral agents with activity against *C. glabrata*, such as voriconazole, itraconazole, or newer drugs like ibrexafungerp (Brexafemme) or oteseconazole (Vivjoa), may be considered if available. Repeat swabbing as a “test of cure” may be helpful, as prolonged symptoms despite a negative posttreatment swab may indicate an alternative or additional diagnosis to VVC.

Abbreviations: RVVC, recurrent vulvovaginal candidiasis; VVC, vulvovaginal candidiasis.

**Figure 2.**
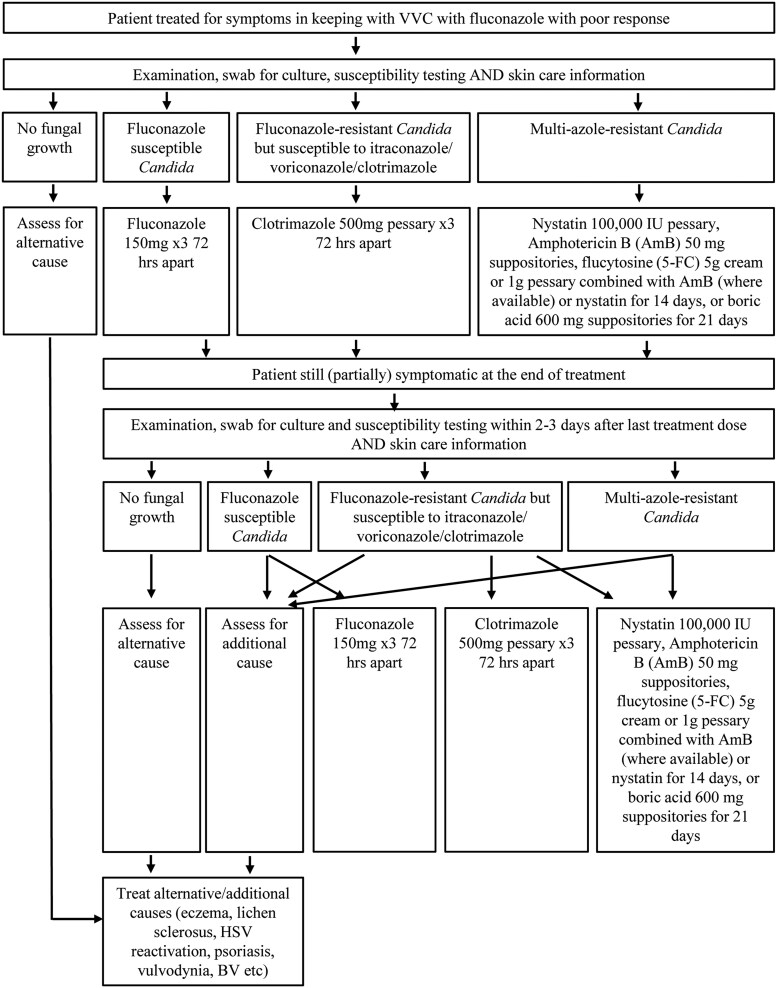
Clinical decision-making process for patients presenting with VVC and recurrent VVC symptomatology. Abbreviation: VVC, vulvovaginal candidiasis.

Isolation of a NAC defined vaginal isolate from symptomatic women with RVVC does not establish causality. This is because NAC organisms in general are less virulent than *C. albicans*, lacking all variety of virulence factors and more likely to reflect colonization only. This is particularly the case for *C. glabrata*, although *C. krusei* and *Candida parapsilosis* can cause typical yeast vulvovaginitis. It is essential to exclude other causes of vulvovaginal symptoms (eg, vulvodynia) before incriminating the NAC organisms. In general, most NAC species are less sensitive to azole drugs and more difficult to eradicate, in particular oral fluconazole. Susceptibility testing for vaginal isolates is infrequently available and breakpoints have never been established that reflect vaginal environment (pH of 4–4.5), so clinical experience and trial and error prevail. After initial failure of topical azoles and fluconazole, specialists attempt oral itraconazole 200–400 mg or topical boric acid 600 mg daily, both for at least 2–3 weeks. Other options include topical amphotericin B (3%–10%) often in combination with flucytosine 17% [83]. There are no published data to date with ibrexafungerp or oteseconazole.

In case of fluconazole resistance, other oral azoles rarely provide better efficacy than topical clotrimazole but come with a number of side effects and risk for drug–drug interactions. Therefore, their use should be guided by susceptibility testing and full medical review.

### Novel Antifungals

Ibrexafungerp is the first oral semisynthetic triterpenoid antifungal that effectively inhibits fungal 1,3-β-D-glucan synthase, leading to fungal lysis. In multiple phase 2 and 3 clinical studies, single-day treatment was shown to be effective for the treatment of an acute sporadic VVC and superior to placebo and consequently earned the Food and Drug Administration (FDA) approval [84–86]. In addition, in a placebo-controlled study in women with recurrent VVC, ibrexafungerp administered on 1 day every month for 6 months significantly reduced VVC recurrence, led to achieving FDA approval [86, 87], and serving as an alternative to weekly fluconazole prophylaxis. Ibrexafungerp demonstrates impressive in vitro activity against multiple azole resistant *Candida* species, and it achieves high vaginal tissue concentration and is particularly active at low pH levels. So, is ibrexafungerp a major advance in treatment of VVC? Time will tell. Ibrexafungerp is a much welcome alternative for women allergic or intolerant to azole agents. The drug is generally safe and well tolerated; however, it is contraindicated during pregnancy or in women of reproductive potential not using effective contraception because animal studies have demonstrated embryo-fetal toxicity [86]. For the vast majority of women with either acute or recurrent VVC, which is usually susceptible to azole antifungals, ibrexafungerp has yet to show clinical advantage.

Oteseconazole (VT-1161) is a tetrazole that inhibits the fungal CYP51 enzyme lanosterol 14-demethylase (ERG11, CYP51) inhibiting ergosterol biosynthesis. Oteseconazole is uniquely designed to have greater selectivity for fungal CYP51 as opposed to human CYP51 enzymes with fewer adverse effects and drug interactions as well as enhanced potency [88, 89]. Oteseconazole is highly active against *Candida* species including fluconazole-resistant vaginal isolates and has an extraordinary long half-life of 138 days, resulting in prolonged effective tissue concentration. Oteseconazole has shown to be highly effective for acute VVC, but is not yet FDA approved for this indication [87, 90, 91]. In a large multicenter study for treatment of acute severe VVC in China at a dose of 600 mg on day 1 and 450 mg on day 2, oteseconazole was superior to a 2-dose regimen of fluconazole (150 mg) in achieving clinical and mycologic care at day 28 (*P* = .0002) with a similar rate of adverse effects and good tolerance [91, 92]. Oteseconazole administered in a weekly dosing regimen for a 12-week period after antifungal induction therapy showed to be effective in preventing VVC recurrence in women with RVVC compared to placebo [91–93]. Based on this study oteseconazole received FDA approval for prevention of RVVC as a 12-week regimen following induction therapy with either fluconazole or a 2-day regimen of oteseconazole [91, 94, 95]. Like ibrexafungerp and fluconazole, oteseconazole is contraindicated in pregnancy and in females of reproductive potential, due to the combination of possible ocular abnormalities in the offspring and its 138-day half-life (U.S. Food and Drug Administration, 2022). All this reduces the numbers of eligible women with RVVC and susceptible *Candida* species for oteseconazole prophylaxis. None of the oral azole antifungals should be prescribed to pregnant women with acute VVC or RVVC. Currently, there are scarce data on successful therapy, and the optimal dosage for a successful treatment remains to be determined.

### The Potential Immunomodulatory Role of Zinc

Recently, zinc supplementation has emerged as a promising therapeutic strategy for VVC caused by *C. albicans* [28]. During infection, *C. albicans* expresses a zinc-binding protein Pra1, which enables the yeast to scavenge zinc from its environment, particularly under zinc-limited conditions such as those found in the vaginal mucosa [28, 96]. Pra1 expression is associated with local inflammation and PMN recruitment, key drivers of VVC immunopathology. Because Pra1 facilitates zinc acquisition, its expression is negatively regulated by zinc availability. Zinc supplementation effectively inhibits Pra1 expression, reducing PMN infiltration and inflammation in both in vitro and in vivo models [28]. In a pilot study, 5 of 6 women who applied a zinc-containing vaginal gel for 3 months did not experience recurrence [28]. From a therapeutic perspective, Pra1 represents an attractive target for treating VVC caused by *C. albicans*, with the potential to significantly improve the quality of life of affected women. However, many NAC species, such as *C. glabrata* and *C. krusei*, have lost the PRA1 gene during evolution [28], potentially limiting the effectiveness of zinc-based treatments in these cases and presenting a significant challenge for future therapeutic development. Furthermore, zinc may not be a viable option for treating fungal-bacterial mixed vaginal infections, as it lacks efficacy against bacteria [28] and the PRA1 gene is unique to the fungal kingdom. These findings highlight the urgent need for new therapeutic targets in vaginal infections, despite the promising advancements and an ongoing clinical trial evaluating zinc local gel for the prevention of RVVC in a broader population of women (unpublished data, ClinicalTrials.gov ID NCT05895162).

### Vaccines Against Recurrent Vulvovaginal Candidiasis

RVVC is, in principle, an excellent disease target for vaccination as women affected by RVVC are usually fully immunocompetent for both B- and T-cell immune responses. In addition, investigations in animal models of candidal vaginitis, and observations in women with RVVC, have identified several, pathogenesis-relevant, and protective *Candida* antigens [25, 97–99]. Vaccines made with these antigens are not expected to eliminate the fungus from the vaginal microbiota but to neutralize its pathogenic factors, complementing or even replacing therapeutic agents.

Two subunit vaccines against RVVC have entered randomized, double blind, and placebo controlled clinical trials. The vaccine named NDV-3A (Novadigm Company, US), made of the N-terminal portion of the *C. albicans* adhesin agglutinin-like sequence 3 and aluminum hydroxide as adjuvant, has completed a phase 2 trial in 188 women with RVVC. The data showed a reduction of vaginitis recurrences and lengthening of time to recurrences, particularly in women aged younger than 40 years compared to placebo recipients. The protection was attributed to the rise of anti-Als3 immunoglobulin G antibodies. No safety signals were reported [100, 101].

Another vaccine named PEV7 (Pevion Biotech, Switzerland), which has entered a phase 1, 2A clinical trial, is a virosomal formulation of a recombinant secretory aspartyl proteinase (rtSap2) of *C. albicans* [99]. Initial immunogenicity data in 48 healthy women who received an intramuscular injection with different doses of the vaccine showed the production of neutralizing anti-Sap2-specific immunoglobulin G in serum, vaginal and, prominently, cervical fluid. No safety issues were reported (Pevion announcement from BERN, Switzerland, 11 October 2011 https://www.pevion.com and other unpublished data).

Although neither Novadigm nor Pevion appear to have further progressed into clinical evaluation of their vaccines, likely because of budget limitations, other investigators could build on those initial results.

## CONCLUSION

VVC, both in its acute and recurrent forms, remains one of the most common vaginal infections globally, disproportionately affecting women of reproductive age and exerting a significant burden on quality of life and healthcare systems. Although advances have been made in identifying and understanding the responsible fungal pathogens, especially *C. albicans*, the underlying host–microbe dynamics and the local immune responses remain insufficiently understood. The vaginal environment presents unique research challenges due to its acidic pH, estrogen variations, local microbial diversity, and influence by sociodemographic factors. Current treatment options are often empirical and limited by resistance, drug toxicity, contraindications in pregnancy, and short-lived prevention of recurrence. Emerging therapies, including novel antifungals, zinc-based immunomodulation, and experimental vaccines, offer new promise but also underscore the need for precision approaches grounded in better understanding of local immunity and microbiome. RVVC exemplifies a condition where antifungal therapy alone is insufficient without addressing underlying triggers and ecosystem disruption. The future direction for research and care should prioritize integrated, patient-centered strategies that combine therapeutic innovation with prevention, immune modulation, and not only restoration but maintenance of vaginal microbial homeostasis to prevent infection. Closing these gaps will require sustained investment in translational and multidisciplinary research that considers not only the basic research itself, but also the challenging and exhausting daily experiences of women with VVC episodes.
